# Bioprospecting of a *Metschnikowia pulcherrima* Indigenous Strain for Chasselas Winemaking in 2022 Vintage

**DOI:** 10.3390/foods12244485

**Published:** 2023-12-14

**Authors:** Federico Sizzano, Marie Blackford, Hélène Berthoud, Laurent Amiet, Sébastien Bailly, Frédéric Vuichard, Christine Monnard, Stefan Bieri, Jean-Laurent Spring, Yannick Barth, Corentin Descombes, François Lefort, Marilyn Cléroux, Scott Simonin, Charles Chappuis, Gilles Bourdin, Benoît Bach

**Affiliations:** 1 Oenology Research Group, Department of Plant Production Systems, Agroscope, 1260 Nyon, Switzerland; marie.blackford@agroscope.admin.ch (M.B.); laurent.amiet@agroscope.admin.ch (L.A.); sebastien.bailly@agroscope.admin.ch (S.B.); gilles.bourdin@agroscope.admin.ch (G.B.); 2 Viticulture and Oenology, HES-SO University of Applied Sciences and Arts Western Switzerland, 1260 Nyon, Switzerland; marilyn.cleroux@changins.ch (M.C.); scott.simonin@changins.ch (S.S.); charles.chappuis@changins.ch (C.C.); benoit.bach@changins.ch (B.B.); 3 Ferments Research Group, Department of Development of Analytical Methods, Agroscope, 3003 Liebefeld, Switzerland; helene.berthoud@agroscope.admin.ch; 4 Wine Quality Group, Department of Development of Analytical Methods, Agroscope, 1260 Nyon, Switzerland; frederic.vuichard@agroscope.admin.ch (F.V.); christine.monnard@agroscope.admin.ch (C.M.); stefan.bieri@agroscope.admin.ch (S.B.); 5 Viticulture Research Group, Department of Plant Production Systems, Agroscope, 1009 Pully, Switzerland; jean-laurent.spring@agroscope.admin.ch; 6 Plants and Pathogens Research Group, Geneva School of Engineering, Architecture, and Landscape (HEPIA), HES-SO University of Applied Sciences and Arts Western Switzerland, 1202 Geneva, Switzerland; yannick.barth@hesge.ch (Y.B.); corentin.descombes@hesge.ch (C.D.); francois.lefort@hesge.ch (F.L.)

**Keywords:** non-*Saccharomyces* yeasts, fermentation, flow cytometry, winemaking, microbial communities

## Abstract

Interest in *Metschnikowia* (*M.*) *pulcherrima* is growing in the world of winemaking. *M. pulcherrima* is used both to protect musts from microbial spoilage and to modulate the aromatic profile of wines. Here, we describe the isolation, characterization, and use of an autochthonous strain of *M. pulcherrima* in the vinification of Chasselas musts from the 2022 vintage. *M. pulcherrima* was used in co-fermentation with *Saccharomyces cerevisiae* at both laboratory and experimental cellar scales. Our results showed that *M. pulcherrima* does not ferment sugars but has high metabolic activity, as detected by flow cytometry. Furthermore, sensory analysis showed that *M. pulcherrima* contributed slightly to the aromatic profile when compared to the control vinifications. The overall results suggest that our bioprospecting strategy can guide the selection of microorganisms that can be effectively used in the winemaking process.

## 1. Introduction

Bioprospecting is defined as “the systematic and organized search for useful products derived from bioresources, including plants, microorganisms and animals, that can be further developed for commercialization and overall benefit to society” [1]. As an example, in the field of winemaking, certain non-*Saccharomyces* (*S.*) yeasts (i.e., *Lachancea thermotolerans*) have been isolated, characterized, and made commercially available for use in warm vintages to increase wine acidity by producing lactic acid without the addition of chemicals such as tartaric, malic, or lactic acid [2]. However, the bioprospecting approach can also be used to identify and characterize new microorganisms in an attempt to recreate the aromatic complexity characteristic of certain spontaneous fermentations [3]. Non-*Saccharomyces* yeasts have attracted much interest as producers of aroma compounds [4]. For example, wines produced by *C. zemplinina*/*S. cerevisiae* co-fermentation showed a significant increase in terpenols and a decrease in acetate ester and aldehyde concentrations [5]. Nevertheless, certain species of the genus *Candida* spp. can negatively affect wine quality by producing acetic acid or favoring the growth of acetic bacteria [6,7]. Both the yeast species and the ratio of non-*Saccharomyces/Saccharomyces* genera can influence the organoleptic properties of the final product [8,9]. Therefore, the bioprospecting of non-*Saccharomyces* yeasts is necessary to ensure good wine quality by avoiding potential spoilage problems and providing favorable aromatic characteristics.

Another non-*Saccharomyces* of oenological interest, *Metschnikowia* (*M.*) *pulcherrima*, is usually detected at concentrations ranging from 5% to 40% in grape musts and generally shows low fermentative power [10,11,12]. It has been shown that *M. pulcherrima* has an impact on fermentation due to its high release of aromatic compounds, such as varietal thiols and higher alcohols, and low production of acetate, ethanol, and acids [13,14,15]. In the vinification of Merlot grapes, Varela et al. [16] used the strain AWRI 3050 of *M. pulcherrima* and obtained wines with fruity characteristics compared to those obtained with *S. uvarum*. In white wine vinification, Escott et al. [17] found enhanced aroma characteristics in Airén wines produced by co-fermentations of *M. pulcherrima* with *L. thermotolerans* and *S. cerevisiae*, while Canonico et al. [18] obtained similar results using *M. pulcherrima* and *S. cerevisiae* in Verdicchio fermentations. They showed an improved aroma profile (tropical fruit notes) as well as the bioprotective effect of the *M. pulcherrima* strain used. In this context, the debate on the bioprotective effect of *M. pulcherrima* remains open, as different studies have shown conflicting results, especially regarding the effect of *M. pulcherrima* on *S. cerevisiae* [19,20]. In this paper, we describe the yeast bioprospecting process and the use of an indigenous strain of *M. pulcherrima* in the experimental vinification of the 2022 vintage. For the first time, we vinified the must of Chasselas with *M. pulcherrima*, the most common white grape variety in French-speaking Switzerland, with the aim of discovering whether this non-*Saccharomyces* could contribute to the creation of new aromas and possibly to bioprotection processes.

## 2. Materials and Methods

### 2.1. Isolation of Microorganisms from Vineyards

The sampling campaigns were carried out from May to October 2021. Samples were collected from eight domains (vineyards and wineries) located in four cantons: Genève (GE), Vaud (VD), Neuchâtel (NE), and Valais (VS). Samples were taken from the winery’s equipment and in various locations of the winery by rubbing with sterile swabs. For each winery, 5 samples were taken from the equipment and from different areas of the room. In the vineyard, vine bark, flowers, insects, and whole berries were sampled with disinfected scissors. For each vineyard, 5 samples of each type were collected, for a total of 20 samples. Samples were randomly taken in different locations. All swabs and samples were then placed in sterile 50 mL Falcon tubes and brought to the laboratory. Different concentrations of ethanol (3% and 6%) were added to a liquid YPD-Cm substrate (yeast extract 1% [*w*/*v*], peptone 2% [*w*/*v*], glucose 2% [*w*/*v*], chloramphenicol 150 µg/mL) to pre-select non-*Saccharomyces* and *Saccharomyces* yeasts. Chloramphenicol was used as a bacterial inhibitor. The tubes were then incubated at 22 °C for 48 h. Liquid cultures were then vortexed and diluted in phosphate-buffered saline (PBS; Carl Roth, Arlesheim, Switzerland) from 10^−1^ to 10^−4^, and 75 µL of each dilution was added to Petri dishes (90 mm) containing Wallerstein Laboratory Nutrient (Chemie Brunschwig AG, Basel, Switzerland) agar medium. Petri dishes were incubated at 28 °C for 1 week to allow colony differentiation. Colonies were then isolated from Petri dishes containing approximately 100 colonies. Colonies were selected on the basis of color, shape, and growth rate. All morphologically different colonies were picked from the Petri dish and successively subcultured to obtain pure cultures. Pure yeast cultures were maintained on YPD agar slants at 4 °C and in glycerol stock (15%) at −80 °C for future identification.

### 2.2. Genetic Identification of Isolates

Isolates were freshly plated on YPD agar and incubated at 30 °C for 48 h for DNA extraction. The DNA extraction protocol was adapted from Ripoll et al. [21]. The quantity and quality of the extracted DNA were measured using a UV nano spectrophotometer, NanoDrop 1000 (ThermoFisher Scientific AG, Basel, Switzerland). The optical density ratios at 260/280 nm (DNA/protein ratio) and 260/230 nm (DNA/organic sugar ratio) higher than 1.7 were considered sufficient for PCR. The D1/D2 domain of the 26S rDNA region was amplified using the Bioline^®^ kit (Meridian Bioscience, Cincinnati, OH, USA) with primers NL1 (5′-GCATATCAATAAGCGGAGGAAAAG-3′) and NL4 (5′-GGTCCGTGTTTCAAGACGG-3′) [22] in a BIOMETRA thermocycler (Labgene Scientific SA, Châtel-Saint-Denis, Switzerland). PCR conditions included an initial denaturation at 95 °C for 1 min, followed by 35 cycles of denaturation at 95 °C for 15 s, annealing at 56 °C for 15 s, and an extension at 72 °C for 12 s terminated by a final extension step of 20 s at 72 °C. The resulting PCR products were visualized using a UV transluminator after electrophoresis in a 1% agarose gel (Carl Roth) in Tris/Borate/EDTA buffer pH 8.0 (TBE, Carl Roth) in an electrophoresis tank (Cosmo Bio Co. Ltd., Tokyo, Japan), along with a 1 kb ladder size marker. Satisfactory PCR products were then purified using the Wizard^®^ kit (Promega AG, Dübendorf, Switzerland) and sequenced using the Sanger method at the Microsynth AG facility (Balgach, Switzerland). The sequences obtained were finally edited using the program FinchTV v. 1.4 (Geospiza Inc., Denver, CO, USA) and identified by searching the NCBI nucleotide database using BLAST (https://blast.ncbi.nlm.nih.gov/Blast.cgi, accessed on 7 May 2022).

### 2.3. Single and Sequential Bench-Scale Fermentations

#### 2.3.1. Single Fermentations

Each strain of *Metschnikowia* spp. isolated was tested under alcoholic fermentation conditions. A colony of each strain of *M. pulcherrima* spp. was picked from a YPD agar plate and inoculated into liquid YPD medium at 28 °C for 3 days. From this preculture, 50 mL of commercial red grape juice (Coop, Bern, Switzerland) was inoculated at 1 × 10^5^ viable cells/mL as determined by OD660 measurement. Fermentations were carried out at 20 °C for 2 weeks before chemical analysis.

#### 2.3.2. Sequential Fermentations

In sequential fermentation experiments, microvinifications were carried out in 500 mL glass bottles sterilized at 121 °C for 15 min. A Chasselas must obtained from the 2021 vintage and frozen at −20 °C in a 3 L bag-in-box was used for fermentations. The must was brought to 4 °C the day before the experiment and then equilibrated in a thermostatically controlled bath at 25 °C. Bottling, yeast inoculation, and sampling were performed under a horizontal laminar flow hood. The bottles containing the must were pasteurized at 60 °C for 20 min in a thermostatic bath and returned to room temperature by cooling with running water. A colony of *M. pulcherrima* (UASWS2926 VBI-A02) was picked from a YPD agar plate, inoculated into liquid YPD medium in sterile 200 mL Erlenmeyer flasks, and expanded for 24 h at 30 °C under orbital rotation at 110 rpm. After viable counts by flow cytometry, the yeast was centrifuged at 3000× *g* for 10 min, resuspended in the experimental must, and inoculated into flasks at a final concentration of approximately 1 × 106 viable cells/mL. *S. cerevisiae* (Lalvin CY3079, Lallemand, Blagnac, France), stored as dry yeast at 4 °C, and was rehydrated and inoculated into the must according to the manufacturer’s instructions at a final concentration of 20 g/hL, corresponding to approximately 2 × 10^6^ viable cells/mL. *S. cerevisiae* was inoculated under the conditions of single fermentation (in triplicate) and co-fermentation with *M. pulcherrima*, 5 days after starting the experiment. Once a day, 3 mL of fermenting must were collected for densitometric evaluation using a DMA 35 portable densitometer; results are shown in Oeschle degrees (Antoon Paar, Graz, Austria). Fermentations were considered completed after 3 to 5 consecutive negative readings. At the same time, samples were taken for high-performance liquid chromatography (HPLC) determination of the main biochemical parameters.

### 2.4. Cellar-Scale Experiments

Chasselas grapes (about 800 kg) from the Agroscope domain of Pully (Lausanne, Switzerland) were harvested and processed on 15 September 2022. After crushing, the grapes were pressed using a PX3 instrument (Euro-Machines, Fairfield, CA, USA), and the juice obtained (about 700 L) was treated with potassium metabisulphite at 50 mg/L and then with pectinase at a final concentration of 1 g/hL (Trenolin-Opti, Erbsloh, Geisenheim, Germany). A clarification step was also performed by adding bentonite (Electra, Martin Vialatte, Magenta, France) at 47 g/hL. The static settling process lasted 24 h, after which the must was divided into three 100 L stainless steel tanks. As an enrichment strategy, 30 g/hL of diammonium phosphate was added to the must (about 60 mg/L of assimilable nitrogen). The “classique cuve” condition (CC) was inoculated with CY3079 dry yeast at 20 g/hL (Lallemand, France) and rehydrated in water according to the manufacturer’s instructions. The sequential fermentation condition (SF) was inoculated with *M. pulcherrima* cultivated in a previously prepared Chasselas must (about 5 L). On day +4 after *M. pulcherrima* inoculation, the yeast CY3079 was also inoculated in this experimental condition at 20 g/hL. For the “pied de cuve” (PDC) condition, the starter was prepared by harvesting and processing (crushing/pressing, then potassium metabisulfite treatment) 15 kg of grapes one week before the official harvest. The spontaneously fermenting must (with a densitometric value of 40° Oe) was added to the fermenting tank at a ratio of 1:10. Alcoholic fermentation was carried out at a controlled temperature (20 °C) with cooling jackets placed inside the tanks and monitored daily by densitometry using the DMA35. The alcoholic fermentation was considered as finished when, after 5–7 consecutive days of negative densitometric values, the detection of residual sugar was found to be <1 g/L. The wine was subjected to yeast removal and prepared (treatment with clarifying enzymes) for tangential filtration with 0.2 μM filters. Residual malic acid was consumed by malolactic fermentation after inoculation with *O. oeni* (Viniflora Oenos, Christian Hansen, Hørsholm, Denmark). At the end of fermentation (malic acid levels <0.1 g/L), the wines were chemically stabilized with sulfites (50 mg/L) and later physically stabilized by cooling at 1 °C for 1 month. Bottling (March 2023) was preceded by an additional cartridge filtration step at 0.65 and 0.45 μM. Bottles were stored under controlled conditions before analysis (10–12 °C in the dark). 

### 2.5. Flow Cytometry (FCM)

For FCM analysis, 50 μL of fermenting must (for both bench-scale fermentations and cellar-scale experiments) was diluted at a ratio of 1:20 in PBS and stained with 5-carboxy-fluorescein diacetate-acetoxymethyl ester (CFDA-AM; Fisher Scientific, Waltham, MO, USA) at a final concentration of 2 μM, Syto-41 (Fisher Scientific) at a concentration of 0.5 μM, and propidium iodide (PI, Sigma-Aldrich, Taufkirchen, Germany) at a concentration of 0.5 μg/mL. After incubation for 15 min at room temperature, the sample was collected using a MACSQuant 10 analyzer (Miltenyi, Bergisch Gladbach, Germany). Offline analysis of the FCM files was performed using Flow Logic software v. 8.7 (Inivai Technologies, Mentone, Australia). The parameters analyzed included cell count (expressed as the log of live cells/mL) and relative fluorescence intensity of CFDA (expressed as the median fluorescence intensity [MFI]), a general indicator of the metabolic activity of the cell.

### 2.6. Amplicon-Based Sequencing after Cellar-Scale Fermentation

Must samples (50 mL) were taken before and after the yeast inoculation and then at 1/3 (~56°Oe), 2/3 (~25°Oe), and at the end of alcoholic fermentation. The samples were centrifuged at 3000× *g* for 10 min, and the pellets were stored at −20 °C until DNA extraction. They were then washed in 50 mL of saline peptone water (NaCl 0.8% *w*/*v*, casein peptone 0.1% *w*/*v*, pH 7.1) and decanted by centrifugation (5 min, 200× *g* 4 °C). Regarding the supernatant, 10 mL was used for DNA extraction and centrifuged for 30 min at 4000× *g* at 4 °C. Pellets were resuspended in an 800 μL buffer (25 mM EDTA, 0.125 M Tris-HCl pH 8, 2 M NaCl), and cells were disrupted in a Bead Ruptor 12 (OMNI International Inc., Kennesaw, GA, USA) with 0.1 g of 0.1 mm zirconia beads (OPS Diagnostics, Lebanon, NJ, USA) for 1 min at high speed. Then, 0.2 mL of cetyltrimethylammonium bromide (CTAB) 10% *w*/*v* was added and mixed before incubation at 60 °C for 30 min. After centrifugation (5 min at 16,400× *g*), 0.7 mL of supernatant was transferred, and 0.5 mL of chloroform/isoamyl alcohol at a ratio of 24:1 was added and centrifuged for 15 min at 16,400× *g* at 4 °C. Finally, 0.2 mL of upper phase was processed on the BioRobot^®^ EZ1 workstation (Qiagen, Hilden, Germany) using the EZ1 DNA Tissue Kit (Qiagen). Genomic DNA was eluted in a volume of 100 μL and the concentration was measured using a NanoDrop^®^ ND-1000 spectrophotometer (NanoDrop Technologies, Thermo Fisher Scientific, Waltham, MA, USA). Amplicon libraries were prepared using the two-step unidirectional fusion method (Thermo Fisher). PCR of the partial translation elongation factor 1-α (TEF1α) was performed in 50-μL reactions using 5 μL of DNA, 0.1 μM of each primer (UnivSeq_EF983F, UnivSeq_983F2, UnivSeq_1567R, and UnivSeq_1576RP, Table 1), 25 μL of Platinum™ PCR SuperMix High Fidelity (Thermo Fisher), and 10 uL of SuperFi GC Enhancer (Thermo Fisher Scientific). Amplification was performed as follows: 98 °C for 30 s, followed by 20 cycles of 98 °C for 15 s, 55 °C for 30 s, 72 °C for 30 s, and a final extension at 72 °C for 5 min. All amplicons were purified using the GeneRead size selection kit (Qiagen). The second PCR step was performed in 25-μL reactions using 2.5 μL of amplicon, 0.1 μM of primer Univ_ABCx, and 0.1 μM of primer Univ_trP1, 12.5 μL of Platinum™ PCR SuperMix High Fidelity, and 5 μL of SuperFi GC Enhancer (Table 1). Amplification was performed as follows: 98 °C for 30 s, followed by 21 cycles of 98 °C for 15 s, 72 °C for 30 s, and a final extension at 72 °C for 5 min. Quality control and quantification of the amplicon library were performed using an Agilent 2100 Bioanalyzer (Agilent Technologies, Santa Clara, CA, USA) and the High Sensitivity DNA assay. All amplicons were then pooled equimolarly, purified using the GeneRead size selection kit, and diluted to a final library concentration of 50 pM. Template preparation, chip loading, and sequencing were performed according to the manufacturer’s instructions using the Ion 520™ and Ion 530™ ExT Kit-Chef and an Ion530 Chip (Thermo Fisher). The raw sequences, were primer trimmed and quality filtered (maxEE = 15, truncQ = 6, maxN = 0, n = 1 × 10^5^, minLen = 100, max-Len = 600) in DADA2. Amplicon sequence variances (ASVs) were obtained in DADA2 with the parameter POOL = “pseudo”. Taxonomic annotation was performed manually using Blast programs developed by Altschul et al. [23].

### 2.7. Must and Wine Analysis

#### 2.7.1. Measurements of Biochemical Parameters by High-Performance Liquid Chromatography (HPLC) and Gas Chromatography (GC)

Sugars, ethanol, glycerol, and acetic, lactic, malic, and tartaric acids were analyzed on an HPLC system (Agilent 1260 Infinity, Agilent Technologies, Santa Clara, CA, USA) equipped with a UV (ultraviolet, G1314F) and a RI (refractive index, 1260 Infinity II) detectors in series. Data acquisition and analysis were performed using the supplied instrument software (Agilent OpenLab CDS Chemstation v.A02.09). The mobile phase consisted of 0.65 mM H_2_SO_4_ and was filtered (0.22 μm, Nylon, Millipore, Burlington, MA, USA) before use. A 500 μL sample was mixed with 4.5 mL of mobile phase and purified using a commercial solid-phase extraction cartridge loaded with 200 mg of sorbent (Waters Oasis HLB 6 cc, Waters AG, Baden-Dättwill, Switzerland). A 20 μL sample was injected and separated at 80 °C on a PS-DVB phase (Aminex HPX-87H, 300 × 7.8 mm, Bio-Rad, Cressier, Switzerland) at a flow rate of 0.5 mL/min. Sugars and glycerol were quantified using a refractive index detector, while acetic, lactic, malic, and tartaric acids were quantified using a UV detector at 210 nm. The method for volatile analysis was adapted from Charapitsa et al. [24]. All chemical standards (acetaldehyde CAS 64-17-5, ethyl acetate CAS 141-78-6, methanol CAS 67-56-1, 2-butanol CAS 15892-23-6, 1-propanol CAS 71-23-8, 2-methylpropanol CAS 78-83-1, 1-butanol CAS 71-36-3, 2-methylbutanol CAS 137-32-6, 3-methylbutanol CAS 125-51-3, ethyl lactate CAS 687-47-8, and 1-hexanol) were provided by Merck (KGaA, Darmstadt, Germany). HPLC-grade ethanol was also purchased from Merck. Deionized water (>18 MΩ) was obtained using the Millipore Treatment System (Millipore). Whiskey control congeners LGC 5100 were purchased from LGC (UK). Sparkling samples were degassed prior to analysis, and samples containing more than 20 g/L sugar were distilled prior to analysis. Samples were transferred to 1.5 mL vials, and 1 microliter was injected into the GC-FID. Standards and blanks were prepared in a water–ethanol solution containing 13% ethanol. All GC separations were performed on an Agilent 7890B gas chromatograph equipped with a 7693 autosampler. High-purity hydrogen was obtained from HG PRO LN (LNI Swissgas, Versoix, Switzerland). The capillary column was an Rt-WAX, 60 m × 0.53 mm i.d, 1.0 um thickness (Restek, Bellefonte, PA, USA), and the injector temperature was set at 100 °C. The oven was programmed at 35 °C for 5 min, increased at 1 °C/min to 65 °C, and then increased at 20 °C/min to 240 °C, followed by 5 min at the final temperature, for a total run time of 48.75 min. The FID temperature was 250 °C. The split ratio was 10:1, and the injection volume was 1 μL. The volatile compound was determined by direct injection. First, a response factor (RF) was calculated for each compound analyzed in a sample using standard solutions. The numerical values of these factors, RF, are calculated from the chromatographic data for standard solutions with known concentrations of the compounds analyzed and can be expressed by the following equation: RRF = ethanol area × standard concentration at 100% ethanol/standard area × ethanol density (789,300 mg/L). The concentration of the sample compound relative to the absolute alcohol was C (mg/L absolute alcohol) = RRF × (unknown area/ethanol area) × ethanol density.

#### 2.7.2. Wine Analysis by Fourier Transform Infrared (FT-IR)

Samples of 30–50 mL of wine after alcoholic fermentation or at the end of the entire winemaking process (bottled wine) were collected and analyzed by FT-IR Wine Scan (Foss, Hillerød, Denmark). Major parameters, such as sugar and acid concentrations, were calibrated against standard methods.

#### 2.7.3. Sensory Analysis

A few weeks after bottling, a sensory profile of the cellar-scale experimental wines was performed by a trained panel of 12 tasters from Agroscope using Redjade software v.5.1.1 (Redjade Sensory Solutions, Martinez, CA, USA). The tasters assessed the intensity of 22 criteria on a scale from 1 (low/poor) to 7 (high/excellent). The three modalities were tasted comparatively; 50 mL of wine was served at 17 ± 1 °C in transparent INAO glasses, anonymized by a three-digit code, and presented in different orders to the panelists. 

### 2.8. Statistical Analysis

Statistical analysis was performed using Prism 10 (Graphpad, Boston, MA, USA). One-way and two-way analyses of variance (ANOVAs) with appropriate post-tests were used.

## 3. Results

### 3.1. An M. pulcherrima Strain Isolated in Vaud Region Produced Low Levels of Acetic Acid/Acetaldeyde and High Levels of 2-Phenylethanol

**Figure 1:**
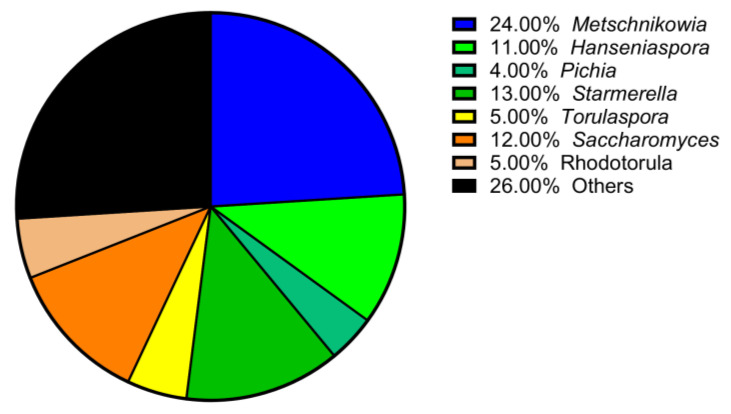
The 7 main yeast genera isolated by bioprospecting in the 4 cantons of French-speaking Switzerland.

After the bioprospecting campaign, seven different yeast genera were isolated. The genus *Metschnikowia* was one of the most abundant, representing 24% of all yeast populations (Figure 1). Molecular analysis identified different species of *Metschnikowia*, namely *M. pulcherrima* (n = 12), *M. reukaufii* (n = 10), *M. fructicola* (n = 4), *M. gruessi* (n = 2), and *M. viticola* (n = 1). One yeast strain could not be identified at the species level. After the isolation and establishment of a biobank, the different *Metschnikowia* strains were tested under fermentation conditions (50 mL) to measure relevant biochemical parameters, including volatile compounds. We chose to set up low-volume fermentations for ease of handling in the lab (number of variants and amount of model must). In particular, it was of interest to find a yeast with low fermentative power that would produce high levels of higher alcohols (e.g., 2-phenylethanol) and low levels of undesirable compounds such as acetic acid or acetaldehyde. Our results showed large variability among the 30 isolates in the production of the compounds of interest, especially acetaldehyde, ethyl acetate, and phenylethanol. However, the fermentative power of the different *Metschnikowia* species was confirmed to be limited among the different species, with a median production of ethanol of about 2.5% (Table 2). At least a dozen isolates of *Metschnikowia* spp. were identified as high producers of 2-phenylethanol (>90 mg/L), and many of them were also high producers of acetaldehyde and ethyl acetate, up to 100 and 500 mg/L, respectively. Overall, no differences in the production of compounds of interest were found among the four main *Metschnikowia* species (i.e., *M. pulcherrima*, *M. reukaufii*, *M. fructicola*, and *M. gruessi*). At the end of the selection process, the best candidate for vinification was found to be a yeast isolated on the domain of the Canton of Vaud. This selected strain, *Metschnikowia pulcherrima* UASWS2926 VBI-A02 (GenBank accession number ON428577), was the highest producer of 2-phenylethanol among the yeasts tested (142 mg/L) and produced low levels of acetaldehyde and ethyl acetate (15 and 35 mg/L, respectively).

### 3.2. M. pulcherrima Is Metabolically Active but Does Not Ferment Sugars in Lab-Scale Co-Fermentations

**Figure 2:**
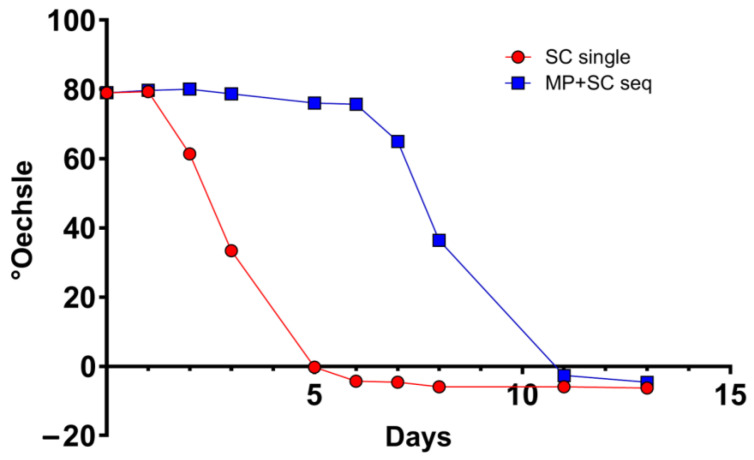
Densitometric analysis of bench-scale fermentations. SC single: *S. cerevisiae* in single fermentation; MP: *M. pulcherrima*; SC seq: *S. cerevisiae* in sequential fermentation.

**Figure 3:**
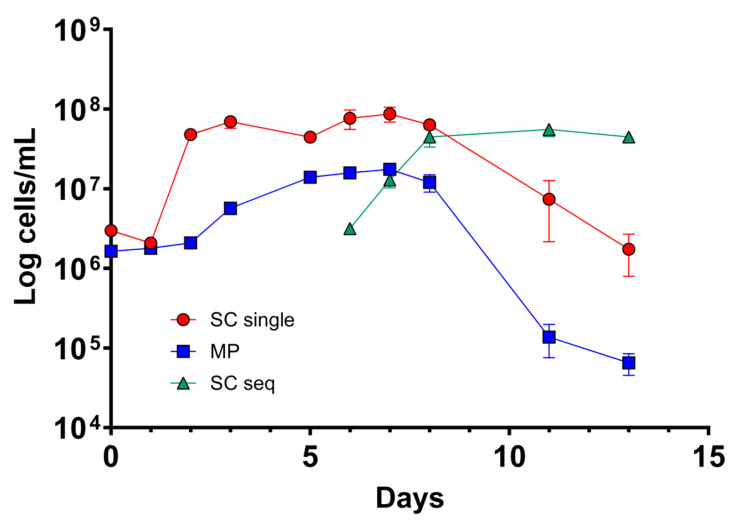
Kinetics of cell growth of *S. cerevisiae* and *M. pulcherrima* (in single or sequential bench-scale fermentation) obtained by FCM analysis. SC single: *S. cerevisiae* in single fermentation; MP: *M. pulcherrima*; SC seq: *S. cerevisiae* in sequential fermentation.

**Figure 4:**
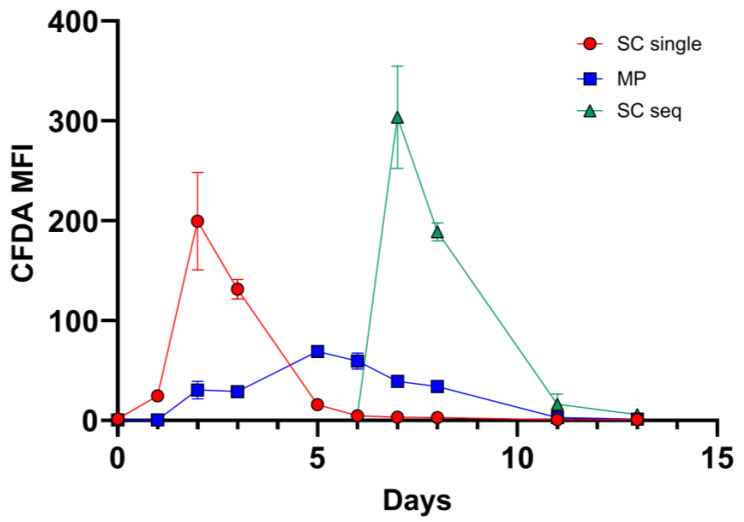
CFDA fluorescence in *S. cerevisiae* and *M. pulcherrima* (in single or sequential bench-scale fermentation) obtained by FCM analysis. SC single: *S. cerevisiae* in single fermentation; MP: *M. pulcherrima*; SC seq: *S. cerevisiae* in sequential fermentation.

To test the compatibility of the selected strain of *M. pulcherrima* with *S. cerevisiae* in sequential fermentation, we carried out a microvinification experiment using a frozen Chasselas must from the 2021 vintage. The oenological parameters of the must before vinification were 90 g/L of glucose, 105 g/L of fructose, 3.6 g/L of malic acid, 5.4 g/L total acidity, 230 mg/L of assimilable nitrogen, and a pH of 3.3. Therefore, in addition to a classical vinification with a commercial *S. cerevisiae*, we prepared a condition in which the *M*. *pulcherrima* UASWS2926 VBI-A02 was present for 5 days before *S. cerevisiae* inoculation. All fermentations were carried out in triplicate. Densitometry showed no significant changes in the condition of *M. pulcherrima* during the first days of incubation (Figure 2). HPLC analysis showed a slight decrease in total sugars (120 g/L at time 0 versus 115 g/L at day 5). Therefore, we performed microbiological analysis using FCM to verify viability and metabolic activity. Our results showed the proliferative activity of *M. pulcherrima* during the first days of incubation, which decreased after inoculation with *S. cerevisiae* (Figure 3). Notably, we were able to discriminate the two co-fermenting yeasts using FCM by analyzing the combination of the metabolic marker CFDA and the nucleic acid marker Syto 41 (Figure S1). The metabolic activity of *M. pulcherrima*, defined by the mean fluorescence of the CFDA marker, followed a similar pattern (Figure 4). The profile of S. *cerevisiae*, both in single and co-fermentation, was similar to that obtained in other microvinifications, namely rapid growth and high metabolic activity defined by the CFDA marker (Figure 3 and Figure 4). To study the influence of the co-fermentation of *M. pulcherrima*/*S. cerevisiae* versus the single fermentation of *S. cerevisiae* on the main biochemical parameters, we performed HPLC analysis. Our results showed that the wine obtained after co-fermentation had significantly lower levels of tartaric acid and higher levels of glycerol than that obtained with single fermentation (Table 3). Overall, these data suggest that the *M. pulcherrima* strain UASWS2926 VBI-A02 could play an active role and thus be used on a larger scale in Chasselas winemaking. Therefore, we prepared an experimental scheme for cellar-scale vinifications of Chasselas in the 2022 vintage.

### 3.3. M. pulcherrima UASWS2926 VBI-A02 in Cellar Assays Recapitulates Bench-Scale Kinetics

**Figure 5:**
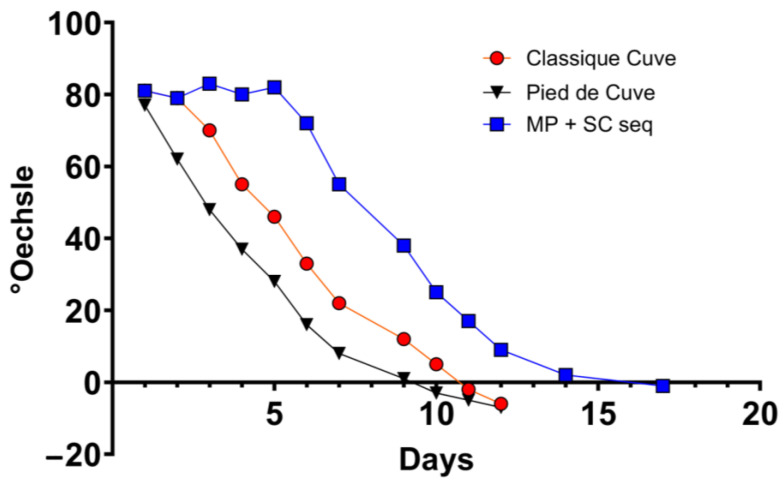
Densitometric analysis of cellar-scale fermentations. MP + SC seq: *M. pulcherrima*/*S. cerevisiae* in sequential fermentation.

**Figure 6:**
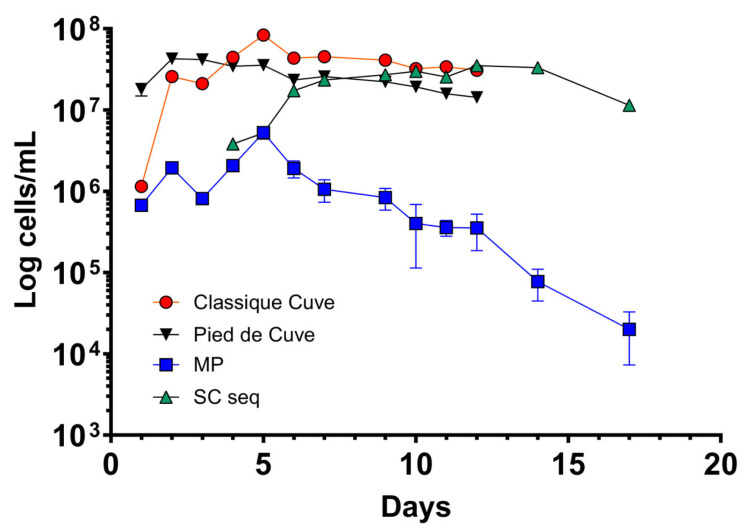
Kinetics of yeast cell growth in the different conditions at cellar scale obtained by FCM analysis. MP: *M. pulcherrima*; SC seq: *S. cerevisiae* in sequential fermentation.

**Figure 7:**
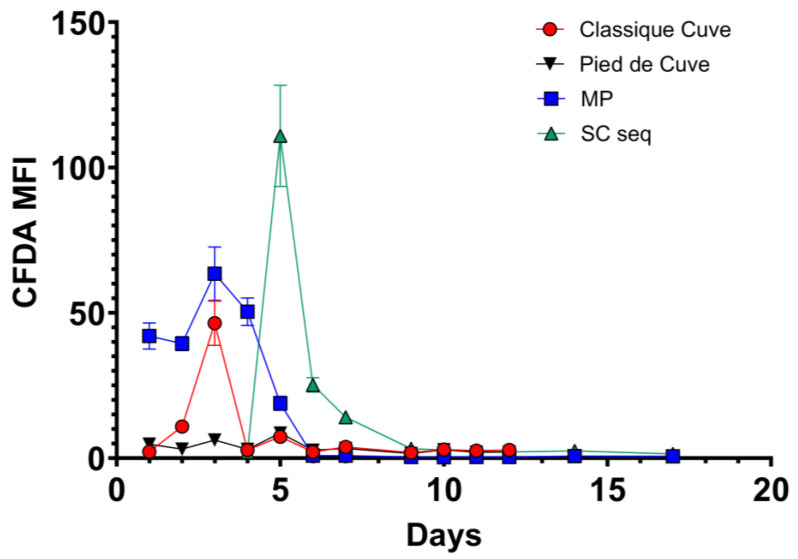
CFDA fluorescence in the different conditions at cellar scale obtained by FCM analysis. MP: *M. pulcherrima*; SC seq: *S. cerevisiae* in sequential fermentation.

For cellar-scale winemaking, three conditions were set up: “classique cuve” (CC; using commercial *S. cerevisiae*), PDC (indigenous fermentation), and SF (using *M. pulcherrima* and *S. cerevisiae*). Even in a larger scale setting, *M. pulcherrima* UASWS2926 VBI-A02 showed little fermentative activity until the co-inoculation, as shown by densitometry data (Figure 5). Again, the FCM results showed that *M. pulcherrima* proliferated and remained viable, albeit at low numbers, until the end of fermentation (Figure 6). Similar to the microvinification results, the metabolic activity of *M. pulcherrima* remained high, at least until inoculation with *S. cerevisiae* (Figure 7). 

### 3.4. The Relative Abundance of Indigenous Microrganisms Decreases after M. pulcherrima UASWS2926 VBI-A02 Inoculum

**Figure 8:**
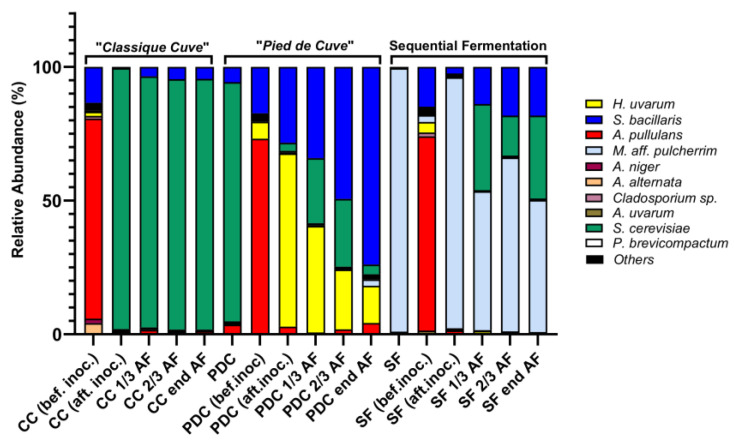
Relative abundance of yeasts in fermentation at different time points in cellar-scale fermentations. Abbreviations: CC (bef. inoc): classique cuve before the inoculation of *S. cerevisiae;* CC (aft. inoc.): classique cuve after the inoculation of *S. cerevisiae;* CC 1/3, 2/3, end AF: classique cuve at 1/3, 2/3, and at the end of alcoholic fermentation; PDC: pied de cuve; PDC (bef. inoc.): condition before the inoculation of the pied de cuve; PDC (aft. inoc.): condition after the inoculation of the pied de cuve; PDC 1/3, 2/3, end AF: pied de cuve at 1/3, 2/3, and at the end of alcoholic fermentation; SF: *M. pulcherrima* only; SF (bef. inoc.): condition before the inoculation of *M. pulcherrima*; SF (aft. inoc.): condition after the inoculation of *M. pulcherrima*; SF 1/3, 2/3, end AF: SF at 1/3, 2/3, and at the end of alcoholic fermentation.

To verify the purity of our *M. pulcherrima* inoculum and to evaluate the presence of other yeast genera/species, we collected molecular data on the composition of the microbial communities present in Chasselas must at different stages of fermentation in different conditions. The average sequencing depth for amplicon sequencing was 121,776 reads (range: 86,178–228,083). In total, 2,070,193 reads were classified to 131 ASVs. All 33 ASVs with a relative abundance greater than 1% were manually identified. Cumulative abundances of these 33 ASVs represented 97.1% to 99.7% of the total relative abundance. Other ASVs were not identified and grouped to the designated name “others” (Figure 8). Our results showed that for the genus *Metschnikowia*, a mixture of two equally represented ASVs was present in the SF condition in a 50:50 ratio. Most likely these were two different copies of the tef1 alpha gene present in the same strain [25]. Interestingly, these two sequences were found to be 100% identical to the *M. citriensis* genome but on two different contigs (ref number Gen Bank: GCA 0097 46055.1). Given the recent incorporation of *M. citriensis* into *M. pulcherrima* [25] and the identification at the isolation stage from the grapevine domains, we were confident that only one strain of *M. pulcherrima* was present in our experiment. It is interesting to note that following inoculation of *M. pulcherrima* UASWS2926 VBI-A02 into the must, the relative abundance of indigenous yeasts such as *Hanseniaspora* (*H.*) *uvarum* or fungi such as *Aureobasidium* (*A.*) *pullulans* decreased. This was evident when compared to the PDC condition. In fact, in the PDC condition, *H. uvarum* and other yeasts persisted together with *S. cerevisiae* throughout the fermentation process (Figure 8).

### 3.5. M. pulcherrima UASWS2926 VBI-A02 Makes Light Aromatic Contributions to Chasselas Vintage 2022

**Figure 9:**
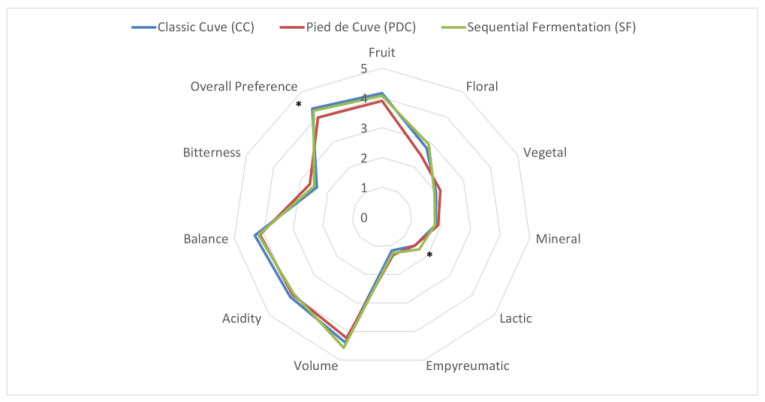
Radar graph showing the results of sensory analysis. For overall preference: * *p* < 0.05 CC vs. PDC; *p* = 0.09 SF vs. PDC; for lactic: * *p* < 0.005 CC vs. SF; *p* < 0.005 PDC vs. SF. Two-way ANOVA with Tukey’s post-hoc test.

The biochemical analysis by FT-IR at the end of alcoholic fermentation did not reveal any differences between the main biochemical parameters (acids, glycerol) among the different conditions, although there was a tendency toward lower acetic acid production in the SF condition, which was not statistically confirmed. Similarly, no significant differences were observed in the parameters analyzed in the finished wine. The sensory analysis of the three wines revealed a slightly more “floral” character of the wine obtained by co-fermentation compared to PDC, although the difference was not statistically significant. On the other hand, there was a statistically significant difference in the perception of a more “lactic” character in the SF wine than in the others. The judges tended to prefer CC and SF wines over PDC (Figure 9). 

## 4. Discussion

Bioprospecting can guide producers in the choice of indigenous yeast strains that can bring new and interesting flavors while reducing the risks of failure that are more common in spontaneous winemaking. In our research, among the eight domains of southwestern Switzerland, we collected a total of seven major genera of indigenous yeasts, with *Metschnikowia* being the most abundant in the sample analyzed. A large number of studies have shown that the diversity of the microbiome (including fungi, yeasts, and bacteria) present in vineyards certainly depends on the location but also on the grape variety and cultivation method [11,26]. The most frequently isolated yeasts were the genera *Hanseniaspora* and *Metschnikowia*, according to Tristezza et al. [27], Belda et al. [28], and Serafino et al. [29], which is congruent with our results. However, a theoretical explanation for the high frequency of *Metschnikowia* spp. found is that the presence of the yeast could be favored by the pressure of *Botrytis cinerea* [30]. In the Swiss vineyards of vintage 2021, after the second half of September, several rains favored the *Botrytis* infection, but due to the low temperatures, the grapes remained healthy [31]. We decided to focus our attention on *Metschnikowia* because it is one of the most interesting genera today from an oenological perspective. To the best of our knowledge, this is the first time that *Metschnikowia* species have been systematically isolated and characterized in southwestern Switzerland. These 30 isolates of the genus *Metschnikowia* were used for a first experimental winemaking at bench scale, and we showed high variability among the oenological parameters of interest, already reported for *M. pulcherrima* species [32], and no significant differences were found among the four main species (*M. pulcherrima*, *M. fructicola*, *M. reukaufii*, *M. gruessii*). The candidate yeast for further experiments had to meet two main criteria: high production of higher alcohols, such as 2-phenylethanol, which gives a “rose” bouquet [33], and low production of compounds that can lead to undesirable aromas when present in high concentrations, such as acetaldehyde [34]. We therefore selected a yeast belonging to the Vaud region that had these characteristics. It should be noted that location was another important factor in the final selection, since proximity of the domain to our analytical laboratories facilitated the study of the ecology of this indigenous strain in subsequent vintages.

Next, we tested the selected strain of *M. pulcherrima* in another microvinification experiment to understand its compatibility with *S. cerevisiae* in co-fermentations and to highlight potential adverse effects. Our results showed that *M. pulcherrima* did not ferment sugars during the first 5 days and that it did not prevent or limit the fermentative power of the *Saccharomyces* after inoculation. However, this result prompted us to test the viability of *M. pulcherrima* in fermentation. We then used FCM to study its physiological state and to rule out the possibility that the Chasselas must used had a negative effect on the viability of the yeast. Our results showed high levels of CFDA fluorescence exhibited by *M. pulcherrima*, and no dead PI-positive cells were detected until at least day 5. In addition, *M. pulcherrima* showed active proliferation until *S. cerevisiae* was added. It is interesting that this double staining, together with the nucleic acid dye Syto-41 and the analysis of the control samples (*M. pulcherrima* fermentations prior to *S. cerevisiae* co-inoculation), allowed us to discriminate the two different types of yeast in co-fermentation. Specifically, we examined the fluorescence intensity of CFDA and Syto 41 in the cell populations at the different sampling points. It should be noted, however, that the absolute specificity of this discrimination method cannot be guaranteed, since these dyes are commonly used in microbiology and are not specific for a given yeast type. The kinetics of the sequential inoculation experiment followed those already described in the literature, with proliferation of *M. pulcherrima* followed by a decrease after inoculation of S. *cerevisiae* [18,35]. Interestingly, our data show that the sequential fermentation had a lower concentration of *S. cerevisiae* compared to single fermentations (inoculated with the same amount of *S. cerevisiae*), even if the metabolic activity as detected by CFDA was comparable. Although *M. pulcherrima* is not considered a high nitrogen-demanding yeast, we could speculate that *M. pulcherrima* UASWS2926 VBI-A02 in the sequential fermentation consumed a certain amount of nitrogen that could have been used by *S. cerevisiae*, resulting in a reduction in the total biomass [36]. At the end of alcoholic fermentation, we then measured the main parameters using HPLC. Our analysis showed a clear increase in the glycerol content in the sequential fermentation and not in the single fermentation, a result that is a general characteristic of sequential fermentation of *M. pulcherrima*/*S. cerevisiae* [35,37,38]. Interestingly, a significant decrease in tartaric acid content without changes in pH (not shown) was observed. This phenomenon, also observed by others [38], remains to be explained. 

After evaluating the feasibility of SF at the bench scale, we moved to the cellar scale to prepare an experiment on Chasselas winemaking with *M. pulcherrima* UASWS2926 VBI-A02. In addition to SF and the single inoculum of *S. cerevisiae* (CC condition), we prepared a PDC condition. Our results at cellar scale generally recapitulate the findings of bench-scale fermentations, especially in cell kinetics, suggesting the appropriateness of our model. Interestingly, in the PDC condition, we did not observe the classical peak of CFDA at the beginning of the fermentation process. Sugar consumption continued, but cell activity was low. Most likely, since they had already undergone many cell divisions, the yeasts in PDC were less active than their “fresh” counterparts (*S. cerevisiae* in CC or SF conditions) but were still able to maintain fermentative activity. In the SF, even if we started with a similar inoculum in terms of cell concentrations (around 1 × 10^6^ cell/mL), we did not observe a similar degree of proliferation of *M. pulcherrima* compared to bench-scale vinifications. For example, at the day of *S. cerevisiae* inoculation, we observed 14 × 10^6^ *M. pulcherrima* cells/mL at bench scale and only 2 × 10^6^ *M. pulcherrima* cells/mL at cellar scale. The day after, the ratio of *M. pulcherrima*/*S*. *cerevisiae* was 5:1 at bench scale, whereas in the cellar scale it was 1:1. This may explain the biochemical differences between the two experiments at the end of alcoholic fermentation, especially the lack of glycerol production in the cellar scale. Thus, at least hypothetically, a low number of *M. pulcherrima* cells were not able to influence *S. cerevisiae* metabolism toward increased glycerol production [39]. The proliferation of *M. pulcherrima* may have been limited by endogenous populations in the must prior to inoculation with *S. cerevisiae*. In fact, after molecular analysis, we found that all the pre-inoculation conditions contained a microbial community that included yeast-like fungi such as *A. pullulans* and other yeasts such as *S. bacillaris* and *H. uvarum*. Interestingly, after inoculation with *M. pulcherrima* UASWS2926 VBI-A02, the relative abundance of these microorganisms was reduced, especially the most “risky” from a spoilage perspective (in particular, they increase volatile acidity), such as *Hanseniaspora*. This bioprotective effect exerted by *M. pulcherrima* is currently being studied extensively from the perspective of a continuous reduction in chemical inputs, such as sulfites [40]. However, it is important to point out that bioprotection is usually carried out in must clarification/maceration steps at low temperatures, conditions that favor biocontrol by *M. pulcherrima* [41,42]. In our experiment, we cannot exclude the effect of an incomplete dilution of the *M. pulcherrima* inoculum in the SF condition (i.e., the presence of a more concentrated *M. pulcherrima* in the sampling volume in the tank even after mixing), since only a short time (approximately 1 h) elapsed between the inoculum and the sampling, making the direct bioprotective effect of *M. pulcherrima* unlikely. Thus, our results are interesting, but the bioprotective effect in the clarified must needs to be studied more thoroughly, with sampling for the determination of microbial communities at precise time points prior to *S. cerevisiae* inoculum. However, the relative abundance does not provide functional information about the microorganisms analyzed (living or dead cells). Therefore, in future bioprotection experiments, it will be essential to complement molecular methods with functional analysis in flow cytometry to obtain the necessary physiological information. At the end of the alcoholic fermentation, stabilization, and filtration phases, the wines were ready for the tasting session by the Agroscope panel. Our results did not show any dramatic change in the Chasselas produced with *M. pulcherrima*, which is important for maintaining the overall sensory expectations of this specific wine. As with most grape varieties, the action of the yeast during alcoholic fermentation can modulate the aromatic profile of Chasselas wine. In this context, we found that the SF conditions imparted a more “floral” character to the wine, although the differences were not statistically significant. We also found a statistically significant difference in the “lactic” character, which was more pronounced in the SF condition. The floral character was described in varieties such as Airén by Escott et al. [17] after the ternary fermentation of *S. cerevisiae* with *M. pulcherrima* and *L. thermotolerans*. To the best of our knowledge, the lactic character has not yet been described in winemaking with *M. pulcherrima,* and it would be interesting to carry out a molecular analysis of the aroma compounds to evaluate, along with the other descriptors, the presence of specific molecules. 

Finally, our results show that the panel’s overall preference was skewed toward the CC and SF rather than the PDC. This is interesting because it suggests that the SF with the chosen *M. pulcherrima* is efficient in creating a wine that is enjoyable and expresses new characteristics without completely changing its original character, resulting in a good alternative to PDC vinifications. However, several aspects remain to be clarified, and additional data need to be collected, especially regarding the bioprotective effect in must after clarification and the *M. pulcherrima*/*S.cerevisiae* ratio in cellar-scale assays. Overall, these data suggest that our bioprospecting strategy is effective in screening and selecting yeasts that can be used at the cellar scale to contribute to the creation of new products in a safe and efficient manner. This is important in a scenario where the use of indigenous microorganisms is becoming increasingly important to wine producers and consumers.

## Figures and Tables

**Table 1:** Primers used in amplicon-based sequencing (cellar-scale experiment).

Primer Name	Primer Sequence
UnivSeq_EF983F	gcagtcgaacatgtagctgactcaggtcacGCYCCYGGHCAYCGTGAYTTYAT
UnivSeq_983F2	gcagtcgaacatgtagctgactcaggtcacGCYCCYGGHCAYAGAGAYTTYAT
UnivSeq_1567R	tggatcacttgtgcaagcatcacatcgtagACHGTRCCRATACCACCRATCTT
UnivSeq_1576RP	tggatcacttgtgcaagcatcacatcgtagACHGTRCCRATACCGGARATCTT
Univ_ABx	CCATCTCATCCCTGCGTGTCTCCGACTCAG|Barcode X|gcagtcgaacatgtagctgactcaggtcac
Univ_ABC01	CCATCTCATCCCTGCGTGTCTCCGACTCAGCTAAGGTAACgcagtcgaacatgtagctgactcaggtcac
Univ_trP1	CCTCTCTATGGGCAGTCGGTGATtggatcacttgtgcaagcatcacatcgtag

**Table 2:** Aggregated values of the enological parameters after microvinification of the 30 isolated *Metschnikowia* strains. 1st Q: first quartile. 3rd Q: third quartile. n.d.: not detected, i.e. below the detection or quantification limit.

	Malic Acid (g/L)	Glucose (g/L)	Fructose (g/L)	Glycerol (g/L)	Acetic Acid (g/L)	Ethanol (%)	Acetaldehyde (mg/L)	Methanol (mg/L)	2-Propanol (mg/L)	1-Propanol (mg/L)	Ethyl Acetate (mg/L)	2-Butanol (mg/L)	2-Methyl-1-propanol (mg/L)	3-Methyl-1-butanol (mg/L)	2-Methyl-1-butanol (mg/L)	Ethyl Butanoate (mg/L)	Ethyl Lactate (mg/L)	2-Phenyl- ethanol (mg/L)
Min	1.580	n.d.	n.d.	2.043	0.062	0.419	0.885	1.894	n.d.	n.d.	0.000	n.d.	0.000	0.000	0.000	0.000	0.000	1.308
Max	2.151	49.048	54.607	4.910	0.356	7.049	94.178	41.002	n.d.	23.004	493.469	n.d.	117.321	105.619	33.290	143.920	57.451	142.353
1st Q	1.674	19.133	33.810	2.667	0.099	1.815	10.463	25.725	n.d.	3.721	34.602	n.d.	32.019	24.374	6.377	85.639	37.043	60.400
Median	1.775	22.352	39.627	3.030	0.119	2.466	17.302	27.715	n.d.	6.747	76.625	n.d.	41.509	36.084	9.888	93.404	44.673	81.973
3rd Q	1.863	29.325	43.146	3.506	0.165	3.150	31.549	33.076	n.d.	9.211	130.171	n.d.	54.256	45.813	12.083	108.174	48.707	96.939
Mean	1.774	24.268	37.812	3.139	0.135	2.452	23.123	27.873	n.d.	6.972	99.272	n.d.	44.600	38.146	10.895	91.890	41.647	77.172
Variance	0.016	106.749	93.930	0.419	0.004	1.567	350.493	54.753	n.d.	29.748	10,271	n.d.	543.457	492.542	54.111	805.333	169.866	961.557
SD	0.125	10.332	9.692	0.648	0.061	1.252	18.721	7.399	n.d.	5.454	101.346	n.d.	23.312	22.193	7.356	28.378	13.033	31.009

**Table 3:** Comparison of oenological parameters after alcoholic fermentation between the single and co-fermentation. * *p* < 0.001 (two-way ANOVA with Sidak test).

	Tartaric Acid (g/L)	Malic Acid (g/L)	Glucose (g/L)	Fructose (g/L)	Acetic Acid (g/L)	Lactic Acid (g/L)	Glycerol (g/L)	Ethanol (%)
SC	3.027 ± 0.101	1.973 ± 0.100	<0.2	<0.2	0.197 ± 0.029	<0.2	3.390 ± 0.191	11.233 ± 0.559
MP + SC	2.527 ± 0.108 *	1.990 ± 0.062	<0.2	<0.2	0.163 ± 0.055	<0.2	4.170 ± 0.173 *	11.320 ± 0.230

## Data Availability

Data are contained within the article and Supplementary Materials.

## Supplementary Materials

The following supporting information can be downloaded at: https://www.mdpi.com/article/10.3390/foods12244485/s1, Figure S1: FCM Gating Strategy.
