# Supplementary material for: Bioprospecting of a Metschnikowia pulcherrima Indigenous Strain for Chasselas Winemaking in 2022 Vintage
Source: Foods. 2023 Dec 14;12(24):4485. doi: 10.3390/foods12244485 (PMC10742927; doi:10.3390/foods12244485)
Supplement: Supplementary file 1 [file foods-12-04485-s001.zip › foods-2730039-supplementary.pdf]

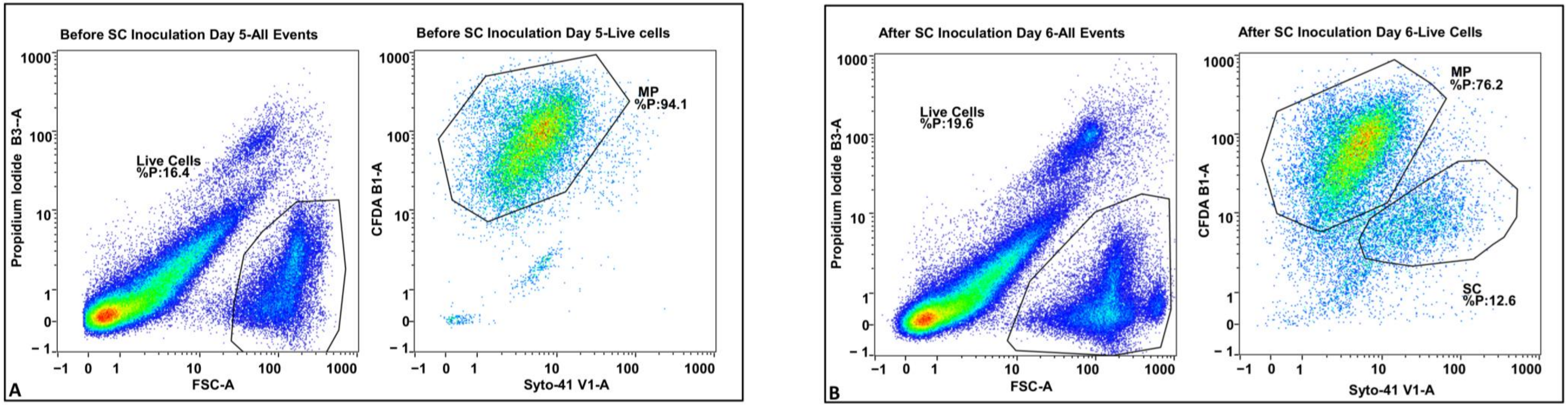

Figure S1. FCM gating strategy. Panel A (before *S. cerevisiae* (SC) inoculation). *M. pulcherrima* (MP) population analyzed by FCM on day 5 of laboratory-scale vinification. Panel B (after SC inoculation on day 5). In addition to the MP population, the population of *S. cerevisiae* is visible. Different colors indicate cell density (blue/green = low cell density; yellow/red = high cell density).
